# Vitamin C Activates Osteoblastogenesis and Inhibits Osteoclastogenesis via Wnt/β-Catenin/ATF4 Signaling Pathways

**DOI:** 10.3390/nu11030506

**Published:** 2019-02-27

**Authors:** Hyeon Kyeong Choi, Gyeong-Ji Kim, Han-Seok Yoo, Da Hye Song, Kang-Hyun Chung, Kwon-Jai Lee, Young Tae Koo, Jeung Hee An

**Affiliations:** 1 Department of Food and Nutrition, KC University, Seoul 077661, Korea; cchk0726@naver.com (H.K.C.); kgj8495@hanmail.net (G.-J.K.); 0223yhs@naver.com (H.-S.Y.); sdh5740@naver.com (D.H.S.); 2 Department of Food Science and Technology, Seoul National University of Science & Technology, Seoul 01811, Korea; carl@seoultech.ac.kr; 3 Department of Biomedical Engineering, Sogang University, Seoul 04107, Korea; 4 Department of Advanced Materials Engineering, Daejeon University, Daejeon 34520, Korea; jmul@ssu.ac.kr; 5 Kwang-Dong Pharmaceutical Co, Ltd., Seoul 06650, Korea; totojazz@naver.com

**Keywords:** Vitamin C, osteoblasts, osteoclasts, osteoporosis, ovariectomized rats

## Abstract

This study evaluated the effects of vitamin C on osteogenic differentiation and osteoclast formation, and the effects of vitamin C concentration on bone microstructure in ovariectomized (OVX) Wistar rats. Micro-computed tomography analysis revealed the recovery of bone mineral density and bone separation in OVX rats treated with vitamin C. Histomorphometrical analysis revealed improvements in the number of osteoblasts, osteoclasts, and osteocytes; the osteoblast and osteoclast surface per bone surface; and bone volume in vitamin C-treated OVX rats. The vitamin C-treated group additionally displayed an increase in the expression of osteoblast differentiation genes, including bone morphogenetic protein-2, small mothers against decapentaplegic 1/5/8, runt-related transcription factor 2, osteocalcin, and type I collagen. Vitamin C reduced the expression of osteoclast differentiation genes, such as receptor activator of nuclear factor kappa-B, receptor activator of nuclear factor kappa-B ligand, tartrate-resistant acid phosphatase, and cathepsin K. This study is the first to show that vitamin C can inhibit osteoporosis by promoting osteoblast formation and blocking osteoclastogenesis through the activation of wingless-type MMTV integration site family/β-catenin/activating transcription factor 4 signaling, which is achieved through the serine/threonine kinase and mitogen-activated protein kinase signaling pathways. Therefore, our results suggest that vitamin C improves bone regeneration.

## 1. Introduction

Osteoporosis is a common skeletal disease characterized by low bone mineral density (BMD) and poor bone quality, which decrease bone strength and increase the risk of fractures [1]. It is the most common multifactorial metabolic bone disorder worldwide and a major public health concern in the elderly and in postmenopausal women [2]. Asians reportedly have the lowest BMD when compared to individuals of African descent, Hispanics, and individuals of European descent [3,4]. According to the Fourth Korea National Health and Nutrition Examination Survey 2008–2009, the prevalence of osteoporosis in Korean adults over 50 years of age was 35.5% in women and 7.5% in men [4]. The more recent 2008–2011 survey reported that the prevalence of osteoporosis in Korea had increased to 38.0% in females aged 50 years and older [5]. 

Increased osteoclast function, decreased osteoblast activity, imbalanced calcium ion (Ca^2+^) metabolism, and the estrogen deficiency-mediated induction of inflammatory diseases may all be involved in the pathology of osteoporosis [6,7]. Dietary and nutritional factors have been identified as having a role in the incidence of osteoporosis and bone fractures. These factors include calcium, vitamin D, phosphorus, caffeine, sodium, dietary protein, and vitamin C [8,9]. Among the nutrients associated with bone formation, vitamin C is known to affect BMD [9]. However, the effects of vitamin C on osteoblastogenesis and osteoclastogenesis in osteoporosis are unclear.

Vitamin C is well-documented as a potent scavenger of free radicals and reactive oxygen species through the provision of hydrogen ions and electrons [10,11]. Additionally, vitamin C is an essential cofactor of prolyl and lysyl hydroxylases, which are key enzymes in collagen biosynthesis [12,13,14]. The potentially beneficial role of vitamin C in preventing low BMD has previously been described [15]. Humans cannot synthesize vitamin C and, therefore, require it as a nutritional supplement [16]. Vitamin C supplementation is especially important in postmenopausal women. Several studies have shown that addition of vitamin C to cultured osteoblast-like cells stimulates the initial deposition of a collagenous extracellular matrix [17,18], followed by the induction of specific genes associated with the osteoblast phenotype, such as alkaline phosphatase (ALP) [18,19] and osteocalcin [18,20], as well as osteopontin, osteonectin, and RUNX2 from undifferentiated mononuclear cells [21,22]. Generally, the ovariectomized (OVX) rat is thought to be a useful animal model for studying the effects of different osteoporosis treatments on the skeletal system [6,7]. Additionally, in OVX mice, vitamin C can prevent the loss of osteoblast differentiation markers (osteocalcin, RUNX2, and bone morphogenetic protein-2 [BMP-2]), attenuate bone loss, and stimulate bone formation [16]. Moreover, vitamin C-deficient mice supplemented with vitamin C display reduced expression of RANKL [23]. 

Recently, the Wnt/β-catenin pathway has been reported to be involved in the differentiation of both osteoblasts and osteoclasts [24,25]. Furthermore, activating transcription factor 4 (ATF4) promotes osteoblast-specific osteocalcin gene expression [26,27]. ATF4 also has a direct and important role in regulating multiple steps of osteoclast differentiation [28]. However, the role of vitamin C in the Wnt/β-catenin/ATF4 pathway has not yet been elucidated.

In the present study, we examined the influence of various doses of vitamin C on bone microstructure and the potential underlying mechanisms by which vitamin C affects bone metabolism in OVX rats. We further investigated the BMP-2/SMAD1/5/8/RUNX2 signaling pathways in osteoblasts and RANK/RANKL and tartrate-resistant acid phosphatase (TRAP) signaling in osteoclasts, which are regulated by Wnt family member 3A (Wnt3a)/β-catenin and mitogen-activated protein kinase (MAPK) signaling pathways. These results provide the first reported evidence that vitamin C regulates both osteoblastogenesis and osteoclastogenesis via the Wnt3a/β-catenin/ATF4 pathways in osteoporosis.

## 2. Materials and Methods

### 2.1. Animals and Diet 

**Figure 1:**
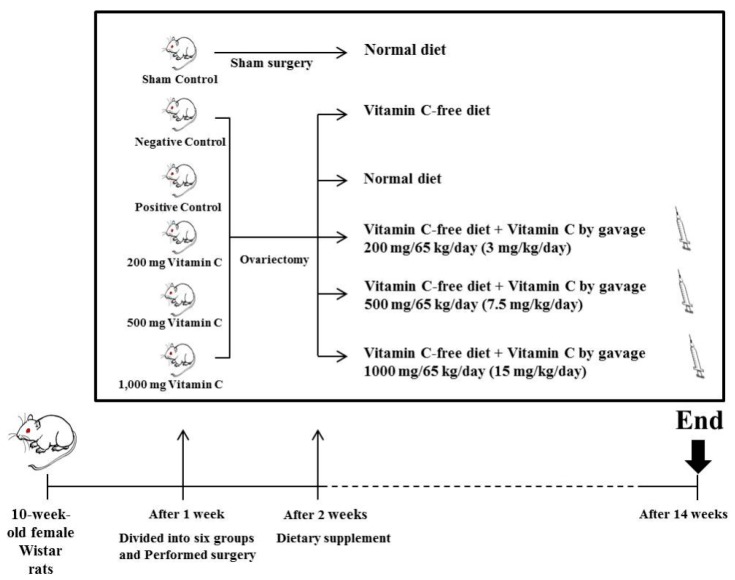
Scheme of animals and diet.

All animal experiments were approved by the Institutional Animal Care and Use Committee of Konkuk University (IACUC approval number: KU 17059). Ten-week-old female Wistar rats, purchased from Doo Yeol Biotech (Seoul, Korea), were housed in a room maintained at 22 °C, with alternating 12-h light–dark cycles. Following a 1-week adaptation period, rats were divided into six groups (*n* = 10 per group) and either ovariectomized (OVX; five groups) or sham-operated (sham; one group, sham surgery, normal diet [TD.97191, Doo Yeol Biotech, Seoul, KR], and 1 mL of distilled water [DW]). Ovariectomy was performed via ligation and excision of the ovaries. Sham surgery involved exposure of the ovaries without excision. After a 1-week acclimatization period, the initial mean rat body weight was 228.78 ± 4.69 g (Table 1). Vitamin C was administered by gavage to relevant groups of rats, once per day. The remaining four OVX groups were fed the following diets: (1) Negative control (OVX, vitamin C-free diet, and 1 mL of DW); (2) positive control (OVX, normal diet, and 1 mL of DW); (3) 200 mg vitamin C (OVX, vitamin C-free diet, and 3 mg/kg vitamin C in 1 mL of DW); (4) 500 mg vitamin C (OVX, vitamin C-free diet, and 7.5 mg/kg vitamin C in 1 mL of DW); and (5) 1000 mg vitamin C (OVX, vitamin C-free diet, and 15 mg/kg vitamin C in 1 mL of DW) (Table 2, Figure 1). Food intake was recorded every day and body weight was measured weekly. At the end of the 12-week feeding period, the rats were sacrificed.

For each animal, tibial bones were dissected and stored at −20 °C. Blood samples were collected from the heart under light anesthesia. Serum was obtained by centrifugation and was stored at −80 °C prior to biochemical assays.

### 2.2. Tibia Bone Ca^2+^ Content 

Tibial Ca^2+^ was quantified using a microwave digestion system (Multiwave 3000; Anton Paar, Graz, Austria) and inductively-coupled plasma mass spectrometry (HP-4500; Hewlett-Packard, Avondale, PA, USA). All tests were performed following the procedures of the Association of Official Analytical Chemists.

### 2.3. Determination of Tibial Bone Strength

The breaking force of the tibia was determined using a three-point bending rheometer (A/WEG wedge fracture probe; Stable Micro Systems, Godalming, UK). The wedge was fractured by the downward motion (3 mm/s) of a 30 mm-wide steel blade. The maximum force (N) applied to break the wedge was used to quantify bone firmness.

### 2.4. Micro-Computed Tomography (Micro-CT) Analysis 

Tibial morphometric parameters were determined in the distal tibia using an Inveon PET high-resolution, cone-beam micro-CT system (Siemens Medical Solutions, Knoxville, TN, USA). Trabecular BMD, cortical BMD, bone surface area/bone volume (BSA/BV), bone volume/total volume (BV/TV), trabecular thickness (Tb.Th), trabecular separation (Tb.Sp), and trabecular number (Tb.N) were determined three-dimensionally by measuring the trabecular bone mass and its distribution. Cortical wall thickness (Ct.Th) was determined three-dimensionally by measuring cortical bone mass and its distribution according to standard procedures. Scans were performed using an applied voltage of 80 kV, with a 1-mm aluminum filter. All cross-sections contained 512 × 512 pixels, with an isotropic voxel size of 9.31 µm. Data were analyzed using the Inveon acquisition workplace software (Siemens). Results were reported according to the published guidelines for the assessment of rodent bone microarchitecture using micro-CT [29].

### 2.5. Histological and Histomorphometrical Analyses of Tibia

Tibias were fixed in 10% neutral-buffered formalin for 2 days at 40 °C. The fixed tibias were decalcified using 10% ethylenediaminetetraacetic acid (pH 7.4), which was replaced daily for 20 days, at room temperature (25 °C). They were then embedded in paraffin blocks and sectioned into 4 μm-thick sections. Some sections were stained with hematoxylin and eosin (H&E), while their adjacent sections were stained for TRAP activity in osteoclasts, using a Leukocyte Acid Phosphatase Assay Kit (Sigma-Aldrich, St. Louis, MO, USA) according to the manufacturer’s instructions. The number of osteoblasts/bone perimeter (N.Ob/B.Pm), osteoblast surface/bone surface (Ob.S/BS), number of osteoclasts/bone perimeter (N.Oc/B.Pm), osteoclast surface/bone surface (Oc.S/BS), number of osteocytes/bone perimeter (N.Ot/B.Pm), and BV/TV were calculated using a bone histomorphometrical analysis program (OSTEOMEASURE™; OsteoMetrics, Decatur, GA, USA).

### 2.6. Reverse Transcription-Polymerase Chain Reaction (RT-PCR) Assay 

Tibial RNA was isolated using TRIzol reagent (Thermo Fisher Scientific, Inc., Waltham, MA, USA). Aliquots (1 μg) of total RNA were reverse-transcribed using SuperScript III Reverse Transcriptase (Invitrogen, Carlsbad, CA, USA). The resulting cDNA was used to determine the tibial mRNA levels of *BMP-2, RUNX2,* osteocalcin, *COL-1*, *RANK*, *RANKL*, *TRAP*, and cathepsin K by PCR amplification using Taq DNA polymerase (KAPA Biosystems, Wilmington, MA, USA). Glyceraldehyde-3-phosphate dehydrogenase (GAPDH) was used as an internal control. Primer sequences were as follows: *GAPDH*: 5’-AACTCCCATTCCACCTT-3’, 5’-GAGGGCCTCTCTCTTGCTCT-3’; *BMP-2*: 5’-AAGGCACCCTTTGTATGTGGACT-3’, 5’-CATGCCTTAGGGATTTTGGA-3’; *RUNX2*: 5’-TCCAGCCACCTTCACTTACAC-3’, 5’-GCGTCAACACCATCATTCTG-3’; osteocalcin: 5’-AGCTCAACCCCAATTGTGAC-3’, 5’-AGCTGTGCCGTCCATACTTT-3’; *COL-1*: 5’-TTGACCCTAACCAAGGATGC-3’, 5’-CACCCCTTCTGCGTTGTATT-3’; *RANK*: 5’-GTGACTCTCCAGGTCACTCC-3’, 5’-GGCAGACACACACTGTCG-3’; *RANKL*: 5’-ACGCAGATTTGCAGGACTCGAC-3’, 5’-TTCGTGCTCCCTCCTTTCATC-3’; *TRAP*: 5’-CGCCAGAACCGTGCAGA-3’, 5’-TCAGGCTGCTGGCTGAC-3’. PCR products were analyzed by 1.2% agarose/ethidium bromide gel electrophoresis and were then photographed.

### 2.7. Western Blotting Analysis

Tibias were dissected free of connective tissue and muscles and homogenized in lysis buffer containing a protease inhibitor (Roche, Mannheim, Germany) and were then centrifuged at 10,000 × g for 10 min at 4 °C. Total protein levels were determined using a protein assay kit (Bio-Rad Laboratories, Hercules, CA, USA). Proteins were subjected to electrophoresis and then transferred onto immobilon-P transfer membranes (Millipore, Burlington, MA, USA). Membranes were blocked with 5% bovine serum albumin prior to incubation with specific primary antibodies against BMP-2, RUNX2, Wnt3a, osteocalcin, COL-1 (Abcam, Cambridge, UK), SMAD1/5/8 (Santa Cruz Biotechnology, Dallas, TX, USA), ATF4 (Boster, Pleasanton, CA, USA), osteoprotegerin (OPG), RANK, RANKL (Bioss Antibodies, Woburn, MA, USA), TRAP, cathepsin K (GeneTex, Irvine, CA, USA), β-catenin, phosphorylated serine/threonine kinase (p-AKT), phosphorylated extracellular signal-regulated kinase (p-ERK), p-p38, phosphorylated c-Jun N-terminal kinase (p-JNK), and β-actin (Cell Signaling Technology, Danvers, MA, USA). The membranes were incubated with either horseradish peroxidase-conjugated goat anti-rabbit IgG (H+L) or goat anti-mouse IgG (H+L) secondary antibodies (Abcam, Cambridge, UK). The antigen–antibody complexes were visualized by enhanced chemiluminescence. Densitometric analysis of the resulting signals was performed using a C-DiGit Blot Scanner (Li-COR, Lincoln, NE, USA).

### 2.8. Statistical Analysis 

Data are presented as the mean ± standard deviation of triplicate experiments. Statistical analyses were performed using SPSS version 18.0 software (SPSS, Inc., Chicago, IL, USA). Comparisons between different groups were carried out by one-way analysis of variance, followed by Duncan’s multiple range post-hoc test. *P*-values < 0.05 were considered statistically significant.

## 3. Results

### 3.1. Food Efficiency Ratio 

Food intake, body weight gain, and food efficiency ratio (FER) did not differ significantly among the experimental groups (Table 1).

### 3.2. Ca^2+^ Content and Tibial Bone Strength are Increased in OVX Rats Treated with Vitamin C

**Figure 2:**
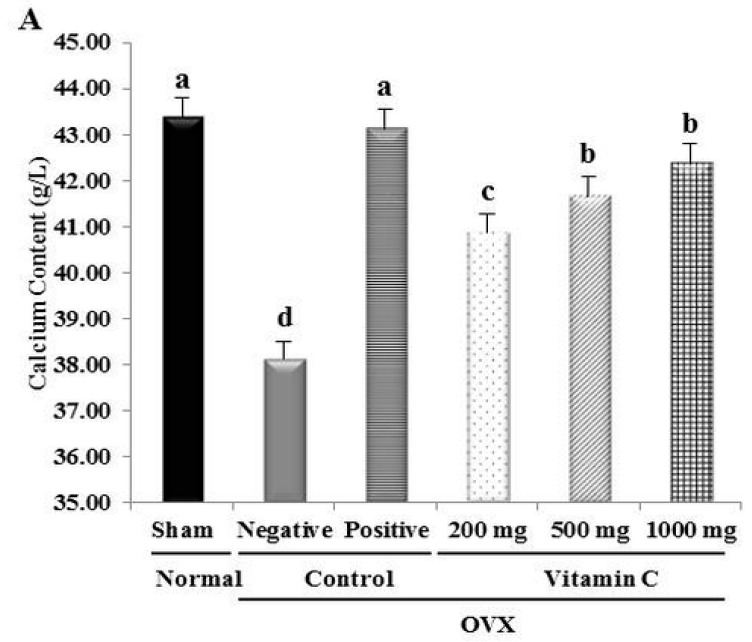

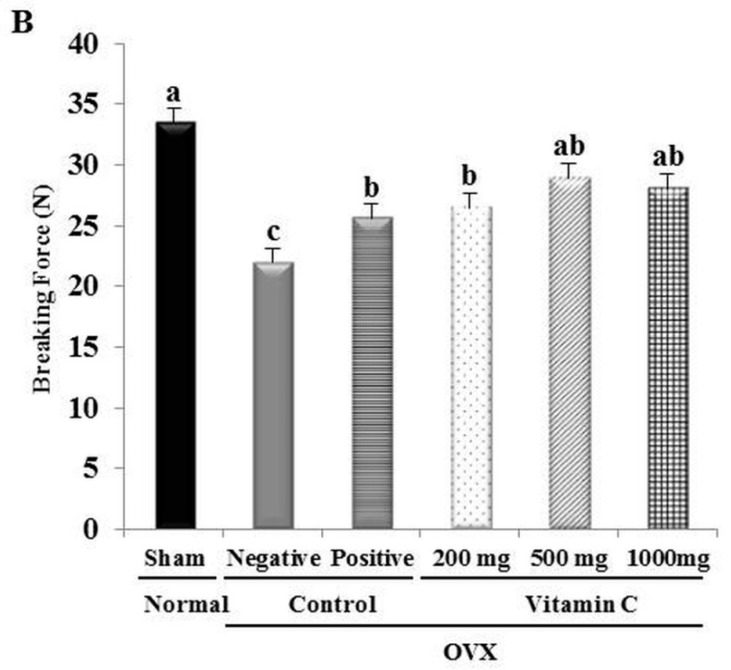
Vitamin C-related increase in calcium content and breaking force in ovariectomized (OVX) rat tibias: (**A**) Calcium content of rat tibias; (**B)** breaking force of rat tibias. Values represent the mean ± standard deviation. Values with different letters were significantly different according to Duncan’s multiple range test (*P* < 0.05).

Tibial Ca^2+^ levels in rats orally treated with vitamin C were all significantly higher than those in negative control tibias. Additionally, the tibial Ca^2+^ content increased with increasing vitamin C concentration (Figure 2). OVX treatment reduced the Ca^2+^ content by 12.1% compared to the sham-operated normal group. Groups treated with 200, 500, and 1000 mg of vitamin C showed significant recovery of Ca^2+^ content by 6.29%, 8.16%, and 9.83%, respectively, compared to the negative control group (Figure 2). Thus, vitamin C recovered the Ca^2+^ content of the tibia in this animal model of osteoporosis.

We tested the effects of vitamin C on the breaking force of the tibial bone using a texture analyzer. Bone strength of the negative control group was significantly lower than that of the sham group and the vitamin C-treated groups (Figure 2). In particular, the breaking energy of the 500 mg vitamin C group was approximately 95% of the breaking energy of the sham group. Additionally, Figure 2B shows that the 200 and 1000 mg vitamin C-treated groups had greater tibial strength than that of the negative control group. These results indicated that vitamin C enhanced the Ca^2+^ content and breaking force of the tibia in OVX rats.

### 3.3. Vitamin C Improves Bone Microarchitecture and Bone Formation Parameters, and Suppresses Bone Resorption Parameters

**Figure 3:**
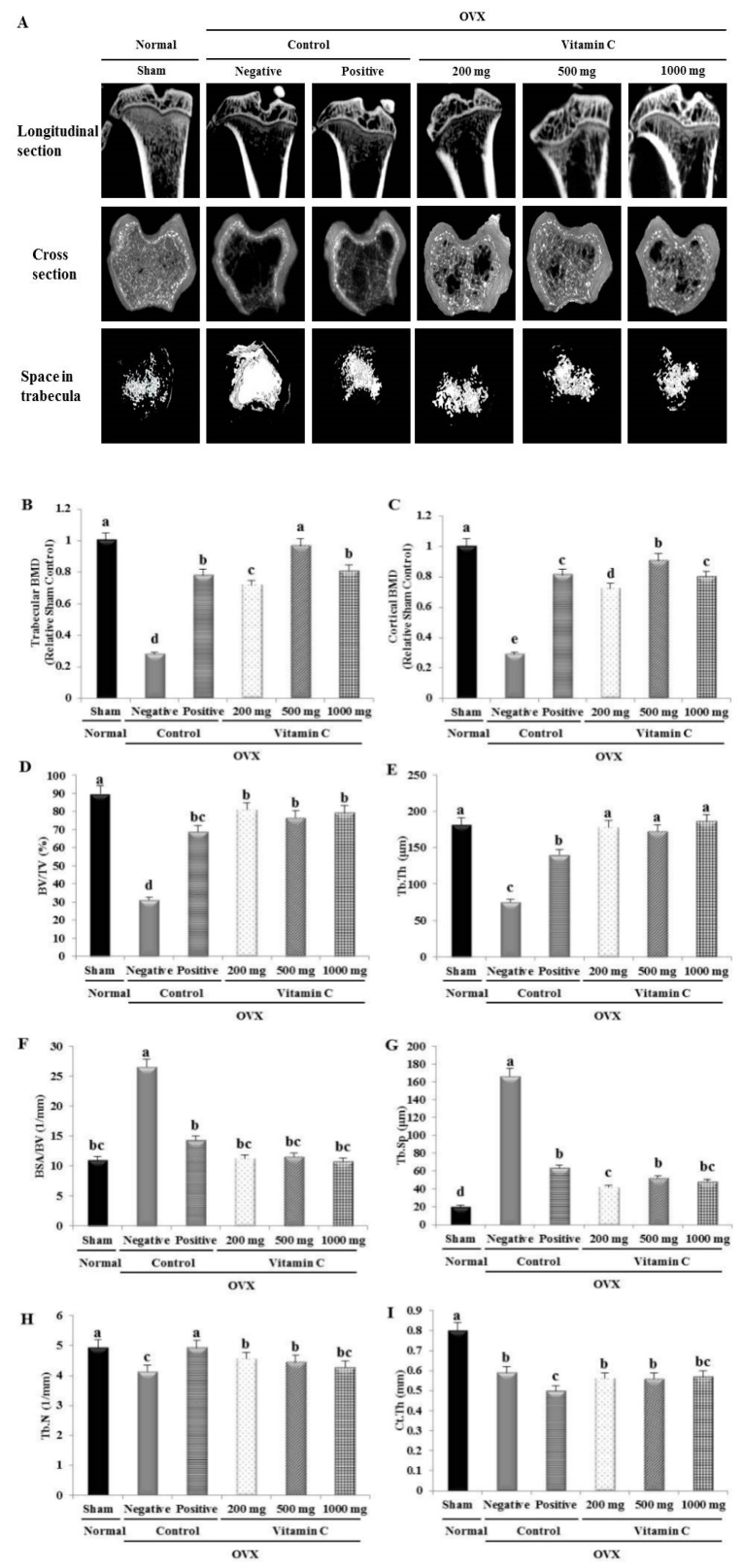
Micro-computed tomography (micro-CT) analysis of the effects of OVX and vitamin C treatment on tibial bone structure: (**A**) Representative image of tibial longitudinal section, cross section, and space of the tibia trabeculae; (**B)** trabecular bone mineral density (BMD); (**C**) cortical BMD; (**D–I**) quantitative analyses of bone volume per total volume (BV/TV), trabecular thickness (Tb.Th), bone surface area per bone volume (BSA/BV), trabecular separation (Tb.Sp), trabecular number (Tb.N), and cortical wall thickness (Ct.Th) of vitamin C-treated tibias. *n* = 10 per group. Values represent the mean ± standard deviation. Values with different letters were significantly different according to Duncan’s multiple range test (*P* < 0.05).

To assess the effect of vitamin C intake on bone metabolism in OVX rats, histological changes in the trabecular structure of the tibia were investigated by micro-CT (Figure 3). There was a large space in the tibial bone because of the decrease in trabecular number, reduced trabecular thickness, and increased trabecular separation in the negative control group (Figure 3). In contrast, in the vitamin C-treated groups, trabecular bone had replaced the empty space in the tibia at 12 weeks (Figure 3). However, there was no significant difference in these parameters according to vitamin C dose.

The values of BMD, BV/TV, and Tb.Th were significantly lower in OVX rats than in sham-operated rats (Figure 3). The trabecular BMD and cortical BMD were increased 3.44- and 3.13-fold, respectively, in the 500 mg vitamin C group compared to the negative control group (Figure 3). Additionally, in the 1000 mg vitamin C group, BV/TV and Tb.Th were nearly 2.5-fold higher than in the negative control group (Figure 3). Furthermore, the BSA/BV and Tb.Sp values in the vitamin C groups were similar to those in the sham group (Figure 3). The Tb.Sp value decreased by 71% in the 1000 mg vitamin C group compared to the negative control group (Figure 3). There were no significant differences in Tb.N among all groups (Figure 3). Ct.Th values in the vitamin C-treated groups were comparable to the value in the negative control group (Figure 3).

### 3.4. Vitamin C Enhances Bone Formation Parameters and Suppresses Bone Resorption Parameters

**Figure 4:**
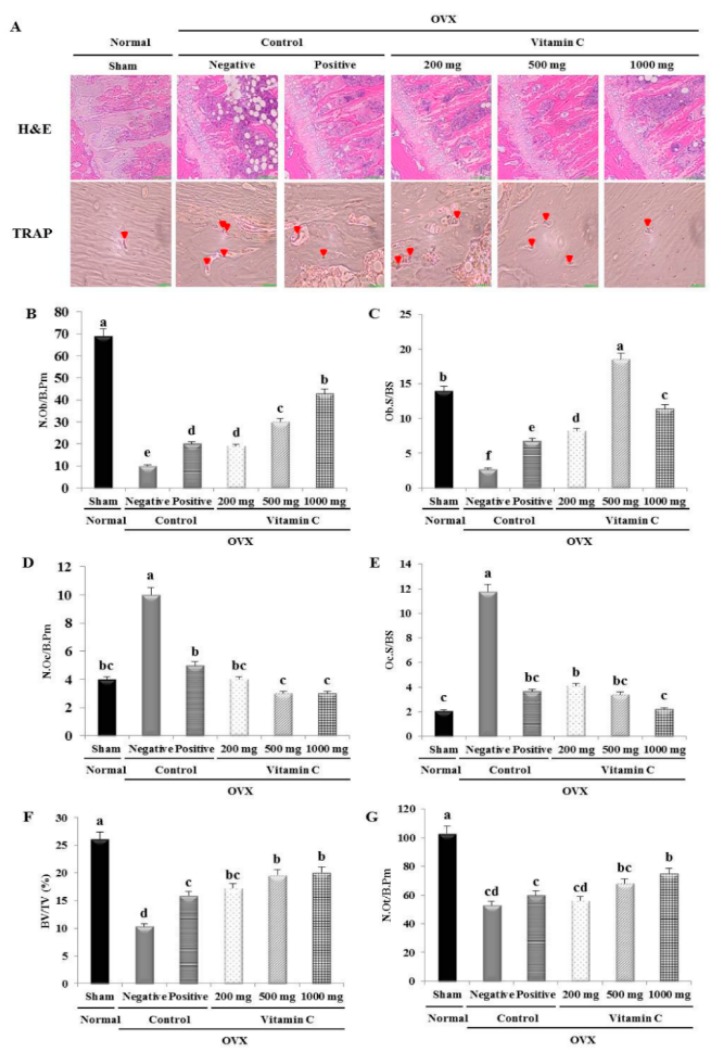
Effect of vitamin C on histomorphometrical analysis in OVX rat tibias: (**A**) Hematoxylin and eosin (H&E) and tartrate-resistant acid phosphatase (TRAP) staining image of the trabecular bone; Dynamic histomorphometrical analyses were performed in this same region to determine (**B**) the number of osteoblasts per bone perimeter (N.Ob/B.Pm), (**C**) osteoblast surface per bone surface (Ob.S/BS), (**D**) the number of osteoclasts per bone perimeter (N.Oc/B.Pm), (**E**) osteoclast surface per bone surface (Oc.S/BS), (**F**) the number of osteocytes per bone perimeter (N.Ot/B.Pm), and (**G**) bone volume per total volume (BV/TV). Data represent the mean ± standard deviation. Values with different letters were significantly different according to Duncan’s multiple range test (*P* < 0.05).

Histomorphometrical analysis of the trabecular bone region in the distal tibia was performed in all experimental groups. H&E staining revealed decreased trabecular bone surface area in the negative control group compared to the sham group (Figure 4). The N.Ob/B.Pm and Ob.S/BS values in the negative control group were significantly lower than in the sham group (Figure 4). Additionally, the negative control group showed increased values for bone resorption parameters, including N.Oc/B.Pm and Oc.S/BS (Figure 4).

According to histomorphometrical analysis, vitamin C treatment caused significant increases in N.Ob/B.Pm and Ob.S/BS values and significant decreases in N.Oc/B.Pm and Oc.S/BS values compared to the values in negative control rats (Figure 4). The group treated with 1000 mg of vitamin C displayed numbers of osteoblasts nearly 4.3-fold higher than the numbers in the negative control group (Figure 4). Furthermore, the Ob.S/BS value of the 500 mg vitamin C group was 6.9-fold higher than the value in negative control rats (Figure 4). In contrast, the Oc.S/BS value in the group treated with 1000 mg of vitamin C was 81.2% lower than the level in the negative control group (Figure 4). In agreement with the micro-CT results, the tibia BV/TV values were significantly higher in the vitamin C groups compared to the negative control group (Figure 3 and Figure 4). No significant differences were observed in the N.Ot/B.Pm values between the OVX groups (Figure 4). These data confirmed that ovariectomy can cause tibial bone loss and that vitamin C can improve the bone microarchitecture of the tibia.

### 3.5. Vitamin C Promotes Expression of Osteoblastogenesis-Related Factors, Including Those in the BMP-2/SMAD1/5/8/RUNX2 Signaling Pathways

**Figure 5:**
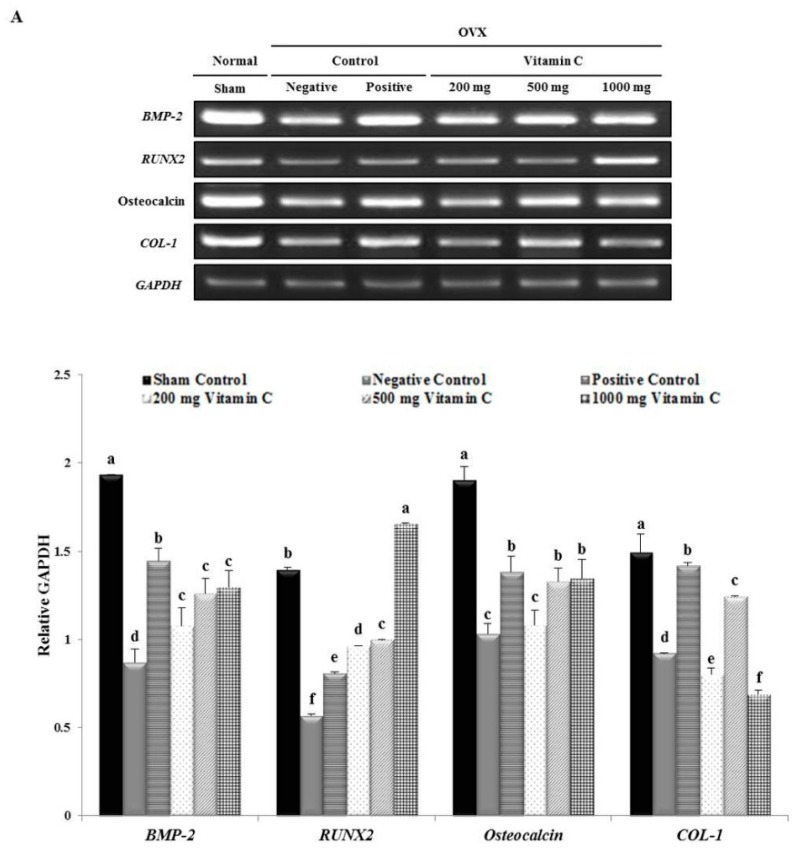

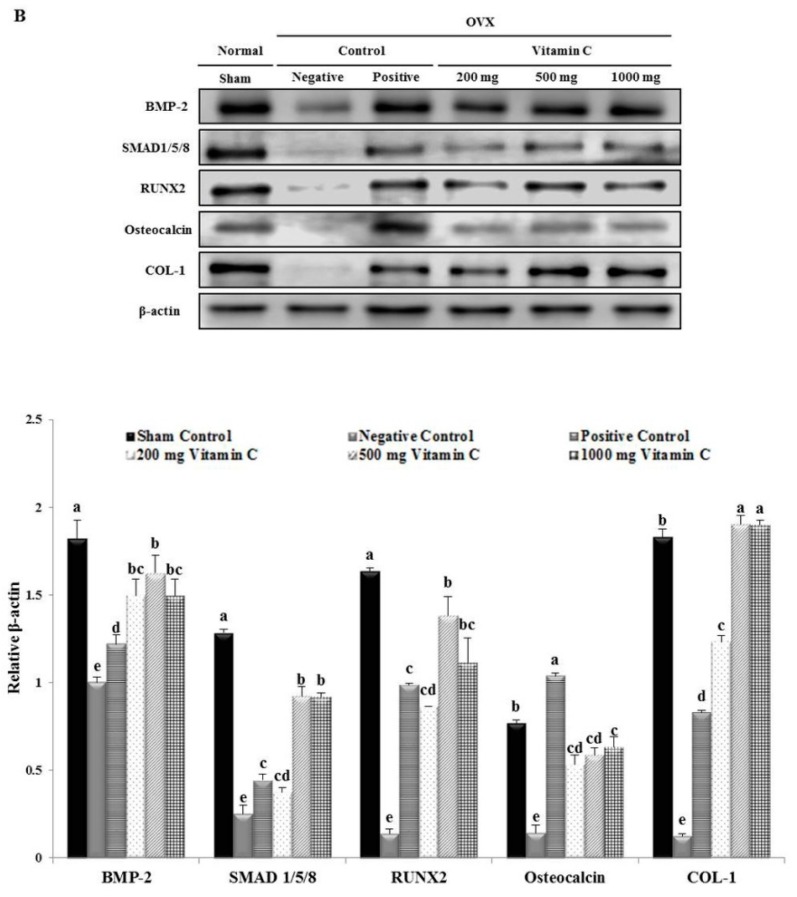
Effect of vitamin C on osteoblast-related gene and protein expression in the tibias of OVX rats. (**A**) Reverse transcriptase-polymerase chain reaction (RT-PCR) products of osteoblastogenesis-related genes. Quantitative assay of mRNA expression levels of *BMP-2*, *RUNX2*, osteocalcin and *COL-1* in vitamin C-treated OVX rats. (**B**) Western blot image of osteoblastogenesis-related proteins and quantitative assay of BMP-2, SMAD 1/5/8, RUNX2, osteocalcin, and COL-1 protein expression in vitamin C-treated rat tibias. Expression was quantified using ImageJ software relative to that of glyceraldehyde 3-phosphate dehydrogenase (GAPDH) and β-actin. Values represent the mean ± standard deviation. Values with different letters were significantly different according to Duncan’s multiple range test (*P* < 0.05).

We examined the impact of vitamin C on bone metabolism indicators, including BMP-2, SMAD 1/5/8, RUNX2, osteocalcin, and COL-1, by RT-PCR and western blot analysis. Expression levels were calculated relative to *GAPDH* and β-actin expression. Vitamin C treatment significantly increased the mRNA expression of osteogenesis-related genes in OVX rats (Figure 5). The mRNA expression levels of *BMP-2* (1.49-fold), *RUNX2* (2.9-fold), and osteocalcin (1.31-fold) in OVX rats treated with 1000 mg of vitamin C were significantly higher than their expression levels in the negative control group (Figure 5). Additionally, the mRNA expression of *COL-1* was 1.35-fold higher in the groups treated with 500 mg of vitamin C than in the negative control group (Figure 5). 

Western blotting analysis showed that ovariectomy significantly decreased the expression of osteogenesis-related proteins, compared to the sham operation (Figure 5). In addition, the vitamin C-treated groups showed increased expression of osteogenesis-related proteins compared to the negative control group (Figure 5). BMP-2 protein expression was increased in OVX rats treated with vitamin C, when compared to rats in the negative control group, but there were no significant differences between groups treated with different doses of vitamin C (Figure 5). The protein expression levels of SMAD 1/5/8 and RUNX2 were higher in the 500 mg vitamin C-treated groups (3.69- and 9.9-fold, respectively) than in the negative control group (Figure 5). The level of osteocalcin protein was dramatically upregulated by treatment with 1000 mg of vitamin C (4.47-fold) compared to the level in the negative control group (Figure 5). Moreover, the protein expression levels of COL-1 in the 200, 500, and 1000 mg vitamin C-treated groups increased by 10-, 15.4-, and 15.4-fold, respectively (Figure 5). These results suggested that oral intake of vitamin C increased the expression of osteoblastogenesis-related genes and proteins in vivo.

### 3.6. Vitamin C Inhibits Expression of Osteoclastogenesis-Related Factors, Including RANK and TRAP

**Figure 6:**
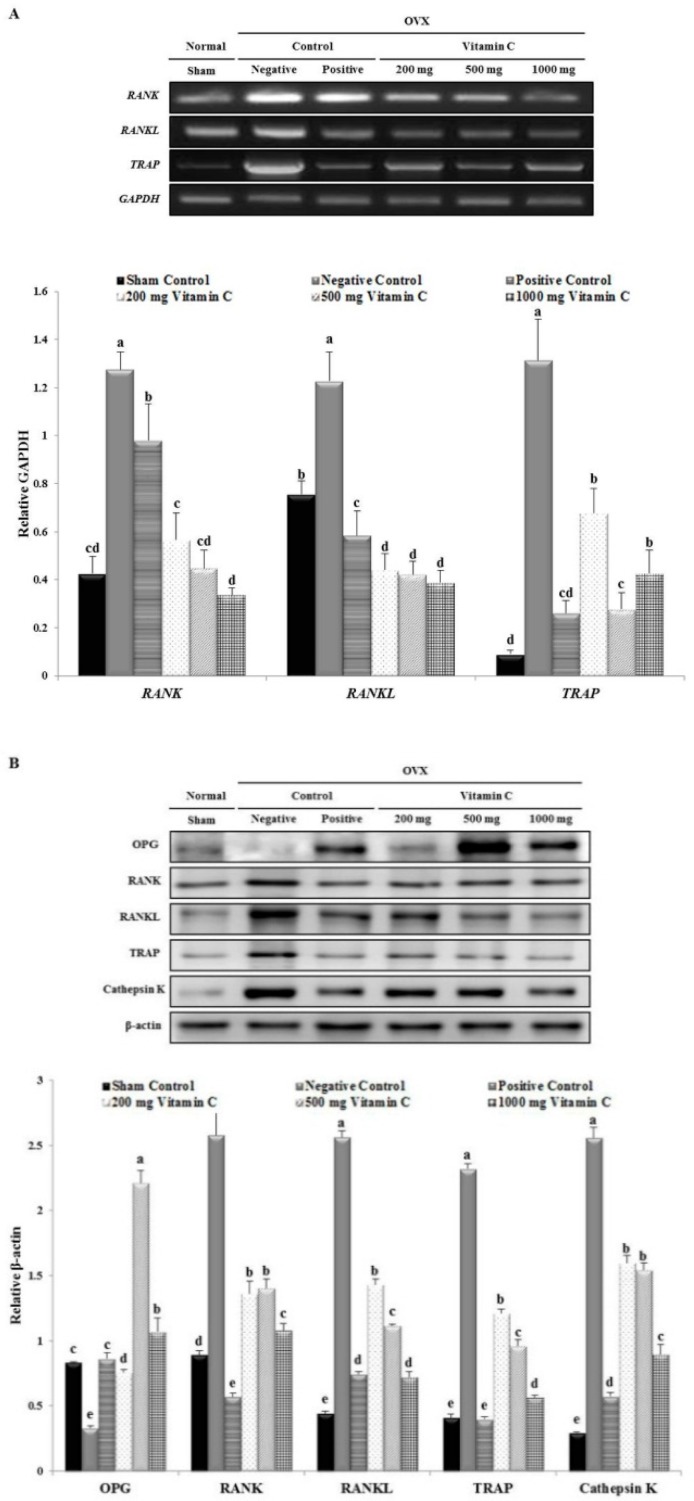
The effect of vitamin C on the expression of osteoclast differentiation-specific genes and proteins in the tibia. (**A**) RT-PCR products of osteoclastogenesis-related genes. Quantitative assay of mRNA expression levels of *RANK*, *RANKL*, and *TRAP* in vitamin C-treated rat tibias. (**B**) Western blot image of osteoclastogenesis-related proteins and the quantitative assay of OPG, RANK, RANKL, TRAP, and cathepsin K protein expression in vitamin C-treated rat tibias. Expression was quantified using ImageJ software relative to glyceraldehyde 3-phosphate dehydrogenase (GAPDH) and β-actin expression. Values represent the mean ± standard deviation. Values with different letters were significantly different according to Duncan’s multiple range test (*P* < 0.05).

Oral intake of vitamin C in rats decreased *RANK*, *RANKL*, and *TRAP* gene expression compared to the levels in the negative control (Figure 6). As shown in Figure 6A, *RANK* mRNA levels in the negative control group were 3.0-fold higher than in the sham group. Meanwhile, *RANK* mRNA levels were 2.3-, 2.8-, and 3.8-fold lower in the 200, 500, and 1000 mg vitamin C-treated groups, respectively, than in the negative control group. Additionally, *RANKL* mRNA was upregulated in the negative control group, while treatment with vitamin C (200, 500, and 1000 mg) inhibited this upregulation (2.8-, 2.9-, and 3.2-fold, respectively) in a dose-dependent manner (Figure 6). These results suggested that vitamin C had a greater attenuating effect than the positive control (Figure 6). *TRAP* mRNA levels in the negative control group were higher than those in all other groups (Figure 6). However, the upregulation of *TRAP* mRNA seen in the negative control group was reversed by vitamin C treatment, showing the maximum effect at a dose of 500 mg.

Figure 6B shows the expression levels of several proteins in vitamin C-treated rat tibias. OPG protein expression was increased by 6.7- and 3.2-fold in the 500 and 1000 mg vitamin C-treated groups, respectively, compared to the levels in the negative control group (Figure 6). Expression of the osteoclast protein, RANK, was significantly increased in the negative control group, but this increase was markedly reversed by 2.4-fold in the 1000 mg vitamin C-treated group (Figure 6). The protein expression level of RANKL was significantly higher (5.78-fold) in the negative control group than in the sham group (Figure 6). However, RANKL protein expression in the vitamin C-treated groups was markedly downregulated (1.8-, 2.3-, and 3.5-fold lower, respectively) compared to its expression in the negative control group (Figure 6). TRAP protein expression increased in the negative control group, but this was significantly reversed by treatment with 200, 500, and 1000 mg of vitamin C (by 1.9- 2.4-, and 4.1-fold, respectively; Figure 6). Furthermore, the negative control showed a significant increase in cathepsin K expression, which was decreased by vitamin C treatment, in a dose-dependent manner (Figure 6). Taking the mRNA and protein expression results together, these data indicated that vitamin C ameliorated the osteoclastic response in osteoporosis.

### 3.7. Vitamin C Regulates Wnt3a/β-catenin, AKT, and MAPK Signaling

**Figure 7:**
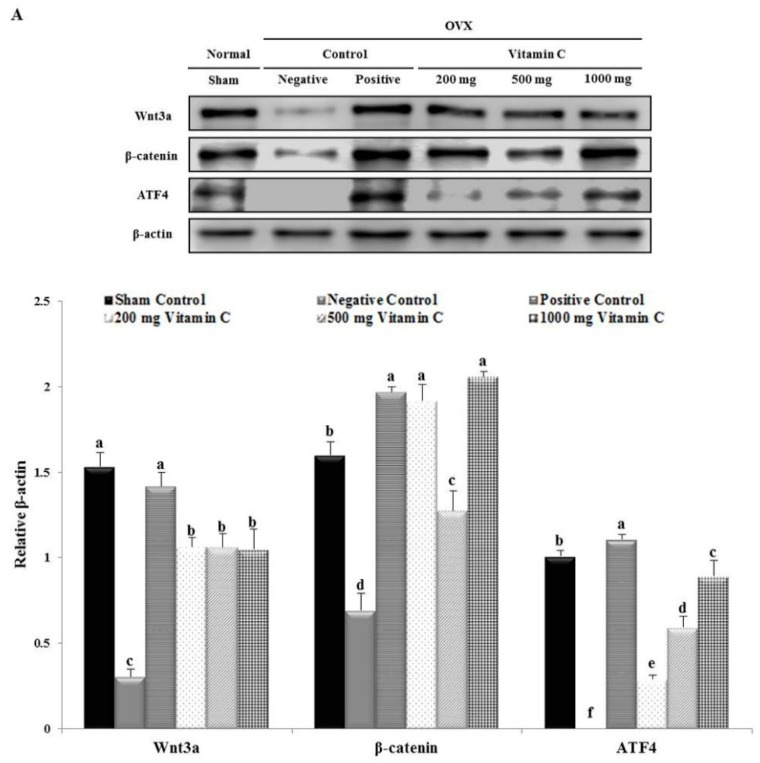

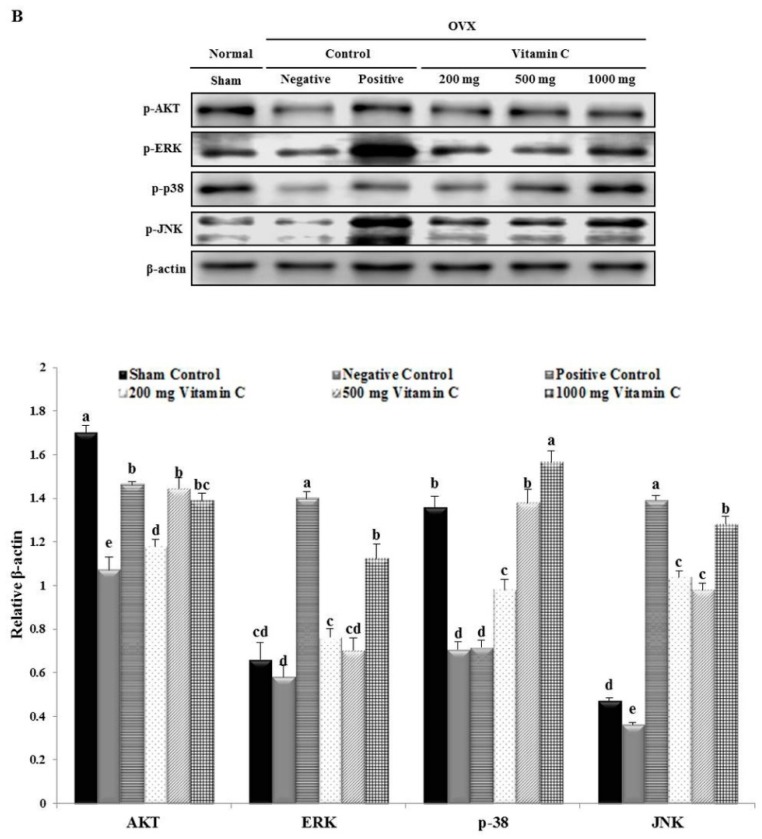
Expression of both osteoblast- and osteoclast-regulated proteins in vitamin C-treated rat tibias. (**A**) Western blot image of Wnt3a, β-catenin, and ATF4 and quantitative assay of Wnt3a, β-catenin, and ATF4 protein expression in vitamin C-treated rat tibias. (**B**) Western blot image of p-AKT, p-ERK, p-p38, and p-JNK and quantitative assay of p-AKT, p-ERK, p-p38, and p-JNK protein expression in vitamin C-treated rat tibias. Expression was quantified using ImageJ software relative to that of β-actin. Values represent the mean ± standard deviation. Values with different letters were significantly different according to Duncan’s multiple range test (*P* < 0.05).

To investigate the mechanism of the effects of vitamin C on osteoblasts and osteoclasts, we performed western blotting analysis of proteins in the Wnt3a/β-catenin signaling pathway (Figure 7). Wnt3a protein expression was not significantly changed by treatment with 200, 500, or 1000 mg of vitamin C (3.51-, 3.49-, and 3.46-fold increases, respectively; Figure 7). The protein expression levels of β-catenin were higher in the 1000 mg vitamin C-treated group (2.98-fold) than in the negative control group (Figure 7). Moreover, the protein expression levels of ATF4 increased at all vitamin C concentrations in a dose-dependent manner (Figure 7).

The expression levels of p-AKT, p-ERK, p-p38, and p-JNK were lower in the negative control group compared to the sham group (Figure 7). However, the decrease in p-AKT protein expression was recovered at all doses of vitamin C treatment (Figure 7). Furthermore, the protein expression levels of p-ERK and p-JNK in the 1000 mg vitamin C-treated group (1.94- and 3.56-fold, respectively) were higher than those in the negative control group (Figure 7). The protein levels of p-38 increased in a dose-dependent manner after treatment with 200, 500, and 1000 mg of vitamin C (Figure 7). These results suggested that vitamin C treatment activated the p-AKT, p-ERK, p-p-38, and p-JNK pathways.

## 4. Discussion

The current therapy for osteoporosis involves the use of antiresorptive agents (bisphosphonates, raloxifene, and denosumab); anabolic agents (teriparatide, abaloparatide, and romosozumab); and nutritional factors, such as protein, calcium, and vitamin D [30]. Our results suggested that vitamin C may also have an important role in osteoporosis therapy. Vitamin C has been shown to be a pivotal modulator of osteoblastogenesis and osteoclastogenesis in osteoporosis models. In this study, vitamin C prevented bone loss and increased the Ca^2+^ content and BMD in the tibias of OVX rats. This is the first study to examine the effects of vitamin C on osteoblastogenesis and osteoclastogenesis, which are regulated by the Wnt3a/β-catenin/ATF4 pathway through MAPK signaling pathways, in an OVX rat model.

Generally, OVX rats show decreased calcium absorption and enhanced bone loss [31]. However, bone is not lost at a uniform rate at all skeletal sites after estrogen depletion in humans [32] and rats [33]. Previous studies have shown that low vitamin C intake negatively affects calcium metabolism [34,35]. Calcium levels in the bone depend on the progression and severity of menopause (estrogen deficiency) and are regulated by several factors, including parathyroid hormone (PTH), calcitriol, and vitamin D [36]. Vitamin D is a particularly effective regulator of calcium levels and it strongly suppresses PTH levels [36,37]. In addition, when serum calcium levels drop, PTH promotes calcium release from bones [38]. Interestingly, vitamin C supplementation is a possible modality to reduce PTH levels, with few side effects [37]. When serum levels of vitamin C are low, calcium-sensing receptors can become resistant to the effects of PTH [37]. Our results for tibial Ca^2+^ levels are consistent with those of previous studies. In the present study, the oral intake of vitamin C restored OVX surgery-induced calcium loss (Figure 2). Additionally, antioxidant vitamins may decrease the risk of osteoporotic fracture by scavenging free radicals and, in turn, reducing oxidative stress in humans and animals [39]. This also supports our result which shows that vitamin C improved bone strength in the OVX rat tibia (Figure 2). Therefore, vitamin C intake may improve tibial Ca^2+^ content and breaking force.

We examined bone structure by micro-CT analysis, which has advantages, because it is a high-resolution, non-destructive, simple, and rapid technique (Figure 3) [40]. Zhu and Cao [16] reported that treatment with 10,000 mg of vitamin C for 8 weeks stimulated the recovery of BV/TV and Tb.N, with no significant effects on BMD and Tb.Sp. These results are similar to our results. However, in the present study, vitamin C enhanced trabecular and cortical BMD and decreased Tb.Sp (Figure 3). Therefore, our results suggested that the administration period is more important for bone regeneration than the dose of vitamin C, above a certain vitamin C concentration, in OVX rats.

In addition, to better understand the cellular mechanisms responsible for bone recovery in OVX rats, we performed histomorphometrical analysis of trabecular bone in the tibia (Figure 4). A previous study has shown that ovariectomy increases the number of osteoclasts and the osteoclast surface per bone surface [41]. Our histomorphometry results were similar. Thus, both micro-CT and histomorphometrical analyses revealed an increase in BV/TV. Interestingly, our histomorphometrical analysis showed that vitamin C also increased the number of osteoblasts and the osteoblast surface per bone surface, but decreased the number of osteoclasts and the osteoclast surface per bone surface, compared to the negative control group (Figure 4). In addition, consistent with previous results, we observed that more osteoclasts were present in the OVX rat bones [42]. However, vitamin C significantly reduced the number of TRAP-positive cells in the tibia (Figure 4). Therefore, the results of our micro-CT and histomorphometrical analyses suggested that the increased bone mass in vitamin C-treated rats resulted from a dramatic increase in the number of osteoblasts and a decrease in the number of osteoclasts.

Previous reports have demonstrated that BMP-2 induces or promotes the expression of RUNX2 and that these transcription factors are essential for osteoblast differentiation and bone formation [43,44,45,46]. In addition, markers of osteoblast differentiation, such as ALP, COL-1, and osteocalcin, are essential for these processes [47,48,49]. Generally, BMP-2 regulates osteoblast differentiation by stimulating osteoblast-related transcription factors, such as RUNX2 [47] and SMAD1, and the closely-related protein, SMAD5, specifically mediates the responses to BMP-2 [50]. Thus, our results showed that vitamin C treatment can upregulate BMP-2, SMAD1/5/8, RUNX2, osteocalcin, and COL-1 gene expression in OVX rats (Figure 5). These results agree with those of previous studies. For example, Valenti et al. observed increased BMP-2 and RUNX2 protein expression in ascorbic acid-treated cells [51]. Therefore, vitamin C does not only promote osteoblast differentiation, but also induces the BMP-2/SMADs/RUNX2/osteocalcin/COL-1 signaling pathway (Figure 5).

Moreover, the differentiation of osteoclasts is regulated by RANKL and OPG, a decoy receptor of RANKL, both of which are secreted from osteoblast-lineage cells, including osteoblasts and osteocytes [52]. Furthermore, cathepsin K is abundant in osteoclasts, where it plays a pivotal role in bone remodeling and resorption [53]. Therefore, our results are consistent with those of previous studies showing that a diet supplemented with 1% calcium [54] or Lycii Radicis Cortex extract [55] reduces TRAP, cathepsin K, and RANK expression in OVX rats. Our results demonstrated that vitamin C suppressed osteoclasts via the RANK/TRAP/cathepsin K signaling pathway (Figure 6).

Wnt/β-catenin and ATF4 are able to modulate bone resorption by regulating the activity of both osteoclasts and osteoblasts [24,25]. Additionally, the Wnt/β-catenin pathway plays a role in regulating bone mass and bone cell function and is involved in cellular responses, such as BMP, strain, and oxygen-related stress [56]. Expression of RANKL contributes to the Wnt/β-catenin pathway-mediated regulation of osteoclastogenesis in bone tissue [57]. Interestingly, ATF4 promotes osteoblast differentiation and osteoblast-specific osteocalcin gene expression [26,27]. Furthermore, previous studies have demonstrated that ATF4 has a direct and important role in regulating multiple steps of osteoclast differentiation [28]. In the present study, the expression of Wnt3a, β-catenin, and AFT4 were increased in OVX rats (Figure 7). Du et al. reported that *Polygonatum sibiricum* polysaccharides could effectively promote the osteogenic differentiation of mouse bone mesenchymal stem cells and suppress osteoclastogenesis through the Wnt/β-catenin signaling pathway [24]. Our results demonstrated that vitamin C inhibited osteoporosis by promoting osteoblast formation and blocking osteoclastogenesis through the Wnt/β-catenin signaling pathway. These results suggested that the Wnt/β-catenin/ATF4 pathway had a more critical role during osteogenesis in the vitamin C-treated groups, through the regulation of BMP-2, RUNX2, SMAD1/5/8, COL-1, and osteocalcin.

Activation of the BMP receptor serine/threonine kinase stimulates the PI3 kinase/Akt pathway and acts as a signaling pathway in BMP-specific Smad functions during osteoblast differentiation [58]. In addition, BMP-2 activates non-canonical MAPK signaling pathways to promote the expression of RUNX2 [59,60]. Calcium supplements derived from *Gallus gallus domesticus* have been shown to promote osteoblast differentiation and mineralization in OVX rats, by regulating BMP-2/RUNX2/SMAD5 via the MAPK signaling pathway [54]. Moreover, many previous studies have suggested the possibility of cross-talk between major signaling pathways, including the MAPK pathway and the Wnt/β-catenin signaling pathway [61,62,63,64,65]. The MAPK signaling pathway may regulate the canonical Wnt/β-catenin pathway by the inactivation of glycogen synthase kinase [65,66]. Caverzasio et al. have also demonstrated that Wnt3a induces the temporary activation of ERK and p38, which regulate ALP activity, suggesting a major role for the MAPKs in the differentiation of mesenchymal cells from osteoprogenitors [62]. Moreover, MAPKs are activated downstream of RANK and mediate the cellular response to RANK stimulation [67]. For example, during osteoclast differentiation, RANKL binds to RANK in osteoclast precursors and differentiating osteoclast cells, resulting in the activation of various intracellular signaling pathways involving ERK and JNK [68]. Moreover, a previous report showed that sesamin can induce osteoblast differentiation by activating the MAPK signaling pathway and can indirectly regulate osteoclast development, through the expression of OPG and RANKL [54,67]. In this study, we showed that vitamin C regulated osteoblasts and osteoclasts through the activation of AKT and MAPK signaling pathways.

## 5. Conclusions

**Figure 8:**
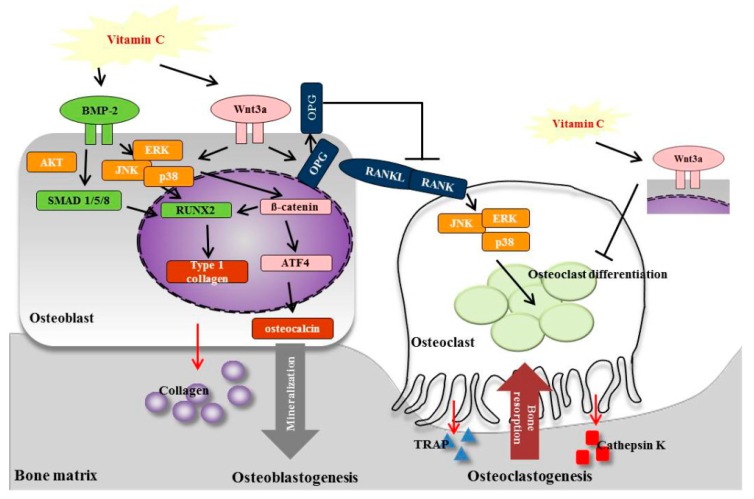
Scheme of vitamin C effects on osteoblastogenesis and osteoclastogenesis signaling pathways.

In conclusion, our data demonstrated that vitamin C enhanced osteoblastogenesis and simultaneously suppressed osteoclastogenesis in vivo. Our results showed that ovariectomy resulted in a failure of normal bone acquisition in rats, via severe deficits in calcium and BMD, which were rapidly recovered by the oral administration of vitamin C in OVX rats, which increased the number of osteoblasts and decreased the number of osteoclasts. The present study also demonstrated that vitamin C enhanced the expression of the osteoblast-specific genes, *BMP-2*, *SMAD1/5/8*, *RUNX2*, osteocalcin, and *COL-1*, in an in vivo model of osteoporosis. Vitamin C also decreased the expression of the osteoclast-specific genes, *RANK*, *RANKL*, *TRAP*, and cathepsin K. Furthermore, vitamin C induced these effects on osteoblasts and osteoclasts via not only MAPK signaling pathways, but also via the Wnt3a/β-catenin signaling pathway (Figure 8). Future studies will investigate the anti-osteoporosis effects of vitamin C, combined with vitamin D or calcium supplementation.

## Figures and Tables

**Table 1:** Body weight gain and food intake by experimental group.

		OVX				
	Normal	Control		Vitamin C		
	Sham *	Negative **	Positive *	200 mg **	500 mg **	1000 mg **
Food intake (g/day)	25.48 ± 3.13 ^a^	23.47 ± 3.04 ^a^	26.36 ± 4.43 ^a^	22.14 ± 4.93 ^a^	24.05 ± 2.44 ^a^	25.28 ± 4.68 ^a^
Initial body weight (g)	229.48 ± 14.28 ^a^	223.34 ± 13.27 ^a^	234.54 ± 14.46 ^a^	224.47 ± 14.63 ^a^	233.81 ± 15.35 ^a^	227.05 ± 11.48 ^a^
Final body weight (g)	302.58 ± 24.61 ^a^	311.99 ± 22.91 ^a^	314.09 ± 20.88 ^a^	336.39 ± 30.46 ^a^	328.96 ± 34.36 ^a^	327.12 ± 20.18 ^a^
Body weight gain (g/week)	6.09 ± 7.15 ^a^	7.38 ± 2.29 ^a^	6.62 ± 1.66 ^a^	9.18 ± 2.39 ^a^	7.92 ± 2.96 ^a^	8.33 ± 1.36 ^a^
FER ^a^	0.039 ± 0.016 ^a^	0.049 ± 0.015 ^a^	0.039 ± 0.009 ^a^	0.053 ± 0.014 ^a^	0.051 ± 0.019 ^a^	0.053 ± 0.008 ^a^

OVX: ovariectomized, * Sham, Positive Control = normal diet, ** Negative Control, Vitamin C groups = vitamin C-free diet, ^a^ FER: Food efficiency ratio = body weight gain (g/week)/food intake (g/week).

**Table 2:** Composition of experimental diets.

Composition		OVX				
	Normal	Control		Vitamin C		
	Sham *	Negative **	Positive *	200 mg **	500 mg **	1000 mg **
Casein (g/kg)	200.0	200.0	200.0	200.0	200.0	200.0
L-Cystine (g/kg)	3.0	3.0	3.0	3.0	3.0	3.0
Sucrose (g/kg)	334.288	334.288	334.288	334.288	334.288	334.288
Corn Starch (g/kg)	313.0	313.0	313.0	313.0	313.0	313.0
Soybean Oil (g/kg)	60.0	60.0	60.0	60.0	60.0	60.0
Cellulose (g/kg)	40.0	40.0	40.0	40.0	40.0	40.0
Mineral Mix (g/kg) ^a^	13.37	13.37	13.37	13.37	13.37	13.37
Potassium Phosphate, Monobasic (g/kg)	11.43	11.43	11.43	11.43	11.43	11.43
Calcium Carbonate (g/kg)	0.6	0.6	0.6	0.6	0.6	0.6
Vitamin Mix (g/kg) ^b^	10.0	10.0 vitamin C - free	10.0	10.0 vitamin C - free	10.0 vitamin C - free	10.0 vitamin C - free
Ethoxyquin, Antioxidant	0.012	0.012	0.012	0.012	0.012	0.012

* Sham Control, Positive Control = normal diet, ** Vitamin C groups = vitamin C-free diet and vitamin C by gavage; Vitamin C 200 mg, 3 mg/kg/day; Vitamin C 500 mg, 7.5 mg/kg/day; Vitamin C 1000 mg, 15 mg/kg/day. ^a^ Mineral Mix (g/kg) - NaCl: 193.7325, C_6_H_7_K_3_O_8_: 575.9615, K_2_SO_4_: 136.1363, MgO: 62.8322, MnCO_3_: 9.163, C_6_H_5_FeO_7_: 15.708, ZnCO_3_: 4.1888, CuCO_3_: 0.7854, KIO_3_: 0.0262, Na_2_SeO_3_·5H_2_O: 0.0262, CrK(SO_4_)_2_·12H_2_O: 1.4399. ^b^ Vitamin Mix (g/kg) - p-Aminobenzoic Acid: 11.0132, Vitamin C, ascorbic acid, coated (97.5%): 101.6604, Biotin: 0.0441, Vitamin B_12_ (0.1% in mannitol): 2.9736, Calcium Pantothenate: 6.6079, Choline Dihydrogen Citrate: 349.6916, Folic Acid: 0.1982, Inositol: 11.0132, Vitamin K3, menadione: 4.9559, Niacin: 9.9119, Pyridoxine HCl: 2.2026, Riboflavin: 2.2026, Thiamin (81%): 2.2026, Vitamin A Palmitate (500,000 IU/g): 3.9648, Vitamin D3, cholecalciferol (500,000 IU/g): 0.4405, Vitamin E, DL-alpha tocopheryl acetate (500 IU/g): 24.2291, Corn Starch: 466.6878.
